# Update of the Preventive Antibiotics in Stroke Study (PASS): statistical analysis plan

**DOI:** 10.1186/1745-6215-15-382

**Published:** 2014-10-01

**Authors:** Willeke F Westendorp, Jan-Dirk Vermeij, Diederik W J Dippel, Marcel G W Dijkgraaf, Tom van der Poll, Jan M Prins, Frederique H Vermeij, Yvo B W E M Roos, Matthijs C Brouwer, Aeilko H Zwinderman, Diederik van de Beek, Paul J Nederkoorn

**Affiliations:** Department of Neurology, Academic Medical Center, P.O. Box 22660, 1100 DD Amsterdam, the Netherlands; Clinical Research Unit (CRU), Academic Medical Center, P.O. Box 22660, 1100 DD Amsterdam, the Netherlands; Department of Neurology, Erasmus MC University Medical Center, P.O. Box Postbus 2040, 3000 CA Rotterdam, the Netherlands; Department of Neurology, Center of Infection and Immunity (CINIMA), Academic Medical Center, P.O. Box 22660, 1100 DD Amsterdam, the Netherlands; Department of Infectious Diseases, Academic Medical Center, P.O Box 22660, 1100 DD Amsterdam, the Netherlands; Department of Neurology, Sint Franciscus Gasthuis, P.O. Box 10900, 3004 BA Rotterdam, the Netherlands; Department of Clinical Epidemiology Biostatistics and Bioinformatics, Academic Medical Center, P.O. Box 22660, 1100 DD Amsterdam, the Netherlands

**Keywords:** stroke, infection, antibiotics, randomized clinical trial, statistical analysis plan

## Abstract

**Background:**

Infections occur in 30% of stroke patients and are associated with unfavorable outcomes. Preventive antibiotic therapy lowers the infection rate after stroke, but the effect of preventive antibiotic treatment on functional outcome in patients with stroke is unknown. The PASS is a multicenter, prospective, phase three, randomized, open-label, blinded end-point (PROBE) trial of preventive antibiotic therapy in acute stroke. Patients are randomly assigned to either ceftriaxone at a dose of 2 g, given every 24 h intravenously for 4 days, in addition to standard stroke-unit care, or standard stroke-unit care without preventive antibiotic therapy. The aim of this study is to assess whether preventive antibiotic treatment improves functional outcome at 3 months by preventing infections. This paper presents in detail the statistical analysis plan (SAP) of the Preventive Antibiotics in Stroke Study (PASS) and was submitted while the investigators were still blinded for all outcomes.

**Results:**

The primary outcome is the score on the modified Rankin Scale (mRS), assessed by ordinal logistic regression analysis according to a proportional odds model. Secondary analysis of the primary outcome is the score on the mRS dichotomized as a favorable outcome (mRS 0 to 2) versus unfavorable outcome (mRS 3 to 6). Secondary outcome measures are death rate at discharge and 3 months, infection rate during hospital admission, length of hospital admission, volume of post-stroke care, use of antibiotics during hospital stay, quality-adjusted life years and costs. Complications of treatment, serious adverse events (SAEs) and suspected unexpected serious adverse reactions (SUSARs) are reported as safety outcomes.

**Conclusions:**

The data from PASS will establish whether preventive antibiotic therapy in acute stroke improves functional outcome by preventing infection and will be analyzed according to this pre-specified SAP.

**Trial registration:**

Current controlled trials; ISRCTN66140176. Date of registration: 6 April 2010.

## Update

### Introduction

Stroke is a leading cause of death worldwide [1]. Infections occur in 30% of stroke patients and are associated with unfavorable outcomes [2, 3]. Preventive antibiotic therapy lowers infection rate in patients after stroke, but the effect of preventive antibiotic treatment on functional outcome after stroke has not yet been investigated [4, 5]. The Preventive Antibiotics in Stroke Study (PASS) is a phase three randomized clinical trial investigating whether the preventive use of the antibiotic ceftriaxone improves functional outcome in acute stroke patients by preventing infections. We previously published the trial protocol and an update of this protocol; we now present the statistical analysis plan (SAP) [6, 7]. This SAP was drafted without knowledge of any of the outcomes by the investigators and randomization code will not be broken before acceptance of the current paper for publication.

### Summary study protocol

PASS is a multicenter prospective, randomized, phase III, open-label, blinded end-point superiority trial (PROBE) of standard care with preventive ceftriaxone treatment compared to standard care without preventive ceftriaxone. Adult patients with stroke (both ischemic and hemorrhagic), a score ≥1 on the National Institutes of Health Stroke Scale (NIHSS) and stroke onset within 24 hours were included [8]. Patients were excluded in case of infection at admission, use of antibiotics within 24 hours before admission, previous hypersensitivity or anaphylaxis to cephalosporins or penicillin, subarachnoid hemorrhage, pregnancy or when death seemed imminent. Patients were randomly assigned to either ceftriaxone at a dose of 2 g, given every 24 h intravenously for 4 days, in addition to stroke-unit care, or standard stroke-unit care without preventive antibiotic therapy. Randomization was performed through ALEA (online software for randomized trials; https://nl.tenalea.net/amc/ALEA/Login.aspx) and is based on a uniform distribution; weight of the arms is equal (1:1). Randomization is stratified according to study center (academic hospital, large non-academic hospital, or small non-academic hospital) and stroke severity (score on NIHSS 1 to 9 or >9) and performed by using random blocks with a maximum block size of 6; blocks of 2, 4 and 6 are made per stratum combination [9]. The study has a PROBE design, which implies that blinding is lost, but only as to treatment. Patient and physician were aware of treatment allocation; however, the assessors of outcome were not. Data were collected on admission, during hospital stay, and at 3 months by standardized case record forms. The primary outcome is functional outcome at 3 months follow-up, as assessed on the mRS during a structured telephone interview by a trained assessor blinded for treatment allocation. Secondary outcomes are death rate at discharge and at 3 months, infection rate during hospital admission, length of hospital admission, volume of post-stroke care, use of antibiotics during hospital stay, quality adjusted life years (QALYs) and costs. Safety outcomes are complications of treatment, Serious adverse events (SAEs) and suspected unexpected serious adverse reactions (SUSARs). In the initial trial protocol, we presented a binary logistic regression analysis on the dichotomized mRS (0 to 2 versus 3 to 6) as primary outcome, requiring a sample size of 3,200 patients, and a proportional odds model in a secondary analysis of the primary end point [9]. Blinded for any of the outcomes, we have changed the primary analysis in PASS from a binary logistic to an ordinal logistic regression on the original mRS, enhancing statistical power. The adapted power analysis showed that with identical assumptions on the clinical effect, using a 0.05 two-sided significance level and 80% study power, 2,550 patients were needed [9]. The analysis of dichotomized mRS data will now be the secondary analysis of the primary end point.

On 23 March 2014, all patients were included and the last follow-up is expected in June 2014. For the complete study protocol and update, we refer to previous publications [6, 7].

### Protocol developments

PASS is registered at current controlled trials ( http://www.controlled-trials.com; ISRCTN: 66140176; date of registration: 6 April 2010). The medical-ethical board of the Academic Medical Center, Amsterdam, approved the protocol on 5 May 2010, and 29 Dutch participating centers were added in the course of the study. Due to the change in primary analysis of primary outcome from a binary logistic approach to an ordinal logistic regression analysis and an expected rate of patients lost to follow-up and/or patients with incomplete data of 5%, the total sample size was reduced from 3,200 patients to 2,550 patients in 2014 [7]. Importantly, no changes were made regarding the primary outcome measurement (that is, the assumed size of the effect on the mRS). This update of the protocol was recently published in this journal [7].

### Statistical analysis plan

#### General analysis principles

The code of the database will not be broken until all efficacy and safety data up to the last patient are included in the database, after data verification and validation are performed, and after the SAP has been accepted for publication. Analysis will be performed by the investigators of the PASS study group (see Acknowledgements section) assisted by a biostatistician of the Academic Medical Centre in Amsterdam.

#### Patient flow diagram

**Figure 1:**
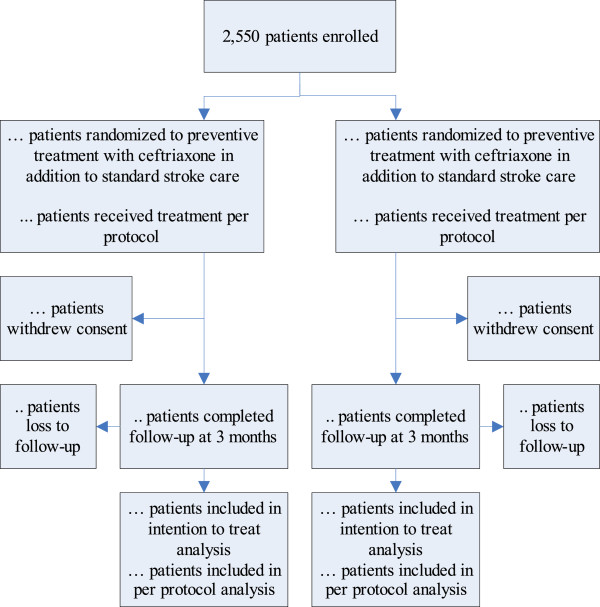
**Flow-chart of patients.**

The flow of participants will be displayed in the Consolidated Standards of Reporting Trials (CONSORT) Flow diagram (Figure 1). Due to the pragmatic design of the study, the total number patients assessed for eligibility has not been assessed.

### Definition of intention-to-treat and per-protocol population

Main analysis will be performed according to the intention to treat (ITT) principle. The safety analysis will be performed in a per protocol (PP) analysis. If a patient was by fault randomized more than once, the first randomization outcome was used. Patients who withdrew consent directly after randomization (that is, before treatment was initiated in those randomized for ceftriaxone in addition to standard care, or within 6 hours after randomization in those randomized for standard care) will be excluded from analysis. Patients with protocol deviations in eligibility are included in the ITT analysis and will be tabulated (Table 1). Patients not receiving their allocated treatment due to instantaneous crossover are considered protocol violations; these patients will be included in the ITT population. PP analysis will exclude patients for whom protocol deviations in treatment and eligibility were made (see protocol deviations in eligibility and protocol deviations in treatment).

**Table 1:** **Number and type of protocol violations in eligibility**

Type of protocol violation in eligibility	Ceftriaxone + standard care (n = …)	Standard care (n = …)
Age <18 years		
Stroke		
No neurological symptoms (NIHSS = 0)		
Onset of stroke >24 hours ago		
Admission		
Infection at admission		
Use of antibiotics <24 hours before admission		
Pregnancy		
Known hypersensitivity to cephalosporins		
Previous anaphylaxis for penicillin derivates		
Subarachnoidal hemorrhage		
Death is imminent		
Total number of protocol violations in eligibility		

### Handling of missing data

If outcome data could not be obtained at the 3 month evaluation, we will first check the municipal council to ensure that the patient is not deceased. All other patients are considered lost to follow-up and will be tabulated, including the percentage of missing outcome data and the association with treatment. Missing outcome data will be obtained by imputation, using the coefficients of five rounds of imputation to obtain the final estimates. We will perform sensitivity analysis. First, we will use single imputation by last observation carried forward (LOCF). An observer blinded for treatment allocation will obtain the last observational score on the mRS using the medical charts and the letters of discharge of the stroke episode. All patients with LOCF will be tabulated with an explanation for the loss to follow-up (Table 2).

**Table 2:** **Assessment of follow-up by LOCF according to treatment allocation**

**Patient number**	**Explanation**	**Treatment allocation**

We will also perform a sensitivity analysis of baseline characteristics of the group of patients not lost-to-follow-up versus all patients included in PASS. In addition, we will also perform a joint model analysis of the loss to follow-up and the mRS change during follow-up [10]. Missing values of baseline characteristics will not be included or imputed in the display of baseline characteristics. When values are missing for dichotomous variables, the actual denominator will be stated. In case of continuous variables, a footnote will be added to show the number of patients for whom the variable was missing.

### Protocol deviations in eligibility, consent procedure, treatment

When a patient was randomized but did not adhere to inclusion or exclusion criteria, this was considered a protocol deviation regarding eligibility. Patients with protocol deviations in eligibility were included in the ITT analysis, but excluded from PP analysis.In each center, the local investigator obtained written informed consent from the patient or representative according to the PASS study protocol. Patients who withdrew consent directly after randomization were excluded from further analysis. The flow of patients is displayed in the CONSORT flowchart (Figure 1).

Treatment allocation was regarded as carried out according to the study protocol when a patient randomized for ceftriaxone in addition to standard care received ceftriaxone 2 gram each 24 hours for 4 days. Patients were also considered as treated PP when treatment was terminated within 4 days due to discharge, death, a palliative care policy, an allergic reaction without anaphylaxis or a previous allergic reaction in medical history (see inclusion and exclusion criteria and protocol deviation in eligibility), other side effects of treatment, or when treatment with ceftriaxone was changed into treatment with another antibiotic because of an infection because these situations and what to do were all defined and described in the initial protocol [6]. In patients allocated to standard care, treatment was carried out according to the study protocol when patients did not receive preventive antibiotic therapy.

### Baseline characteristics

Baseline characteristics of all patients will be outlined per treatment allocation in a baseline table describing the following variables: age, sex, medical history (atrial fibrillation/flutter, stroke, hypercholesterolemia, hypertension, myocardial infarction, cardiac valve insufficiency/stenosis/replacement, peripheral vascular disease, obstructive pulmonary disease, and immunocompromised), current smoking, specific medication (anticoagulants, antiplatelet, statin, ACE inhibitor, ß-blocker, and proton pump inhibitor) prior to stroke, disability prior to stroke on mRS, stroke severity on NIHSS, performance of a screening test for swallowing function, dysphagia, acute treatment (IV thrombolysis and anticoagulant antagonist therapy) and diagnosis at discharge (infarct, haemorrhage, TIA, or other). Outline of the table is displayed in the ‘Outline of figures and tables’ section (Table 3). All variables will be presented categorized by treatment arm. Dichotomous variables will be displayed in percentage with the number of patients divided by the total number of evaluated patients. Continuous variables will be reported as means with standard deviations when normally distributed and in medians with interquartile ranges when they do not meet the criterion of being normally distributed, as assessed by the Kolmogorov-Smirnov test. For continuous variables, the number of patients evaluated will be presented in a footnote of Table 3.

**Table 3:** **Baseline characteristics**

Baseline characteristics	Ceftriaxone + standard care	Standard care
Age - years		
Male sex - % n/N		
Medical history - % n/N		
- Atrial fibrillation/flutter		
- Stroke		
- Hypercholesterolemia		
- Hypertension		
- Myocardial infarction		
- Cardiac valve insufficiency/stenosis/replacement		
- Peripheral vascular disease		
- Obstructive pulmonary disease		
- Immunocompromised		
Current smoker - % n/N		
Medication prior to stroke - % n/N		
- Anticoagulants		
- Antiplatelet		
- Statin		
- ACE inhibitor		
- Bèta-blocker		
- Proton pump inhibitor		
Disability prior to stroke - mRS *		
Stroke severity - NIHSS **		
Swallowing screening performed - % n/N		
Dysphagic patients - % n/N		
Acute treatment - % n/N		
- IV thrombolysis		
- Coagulant therapy		
Diagnosis at discharge - % n/N		
- Infarction		
- Hemorrhage		
- Transient ischemic attack (TIA)		
- Other		

*mRS, denotes modified Rankin Scale.

**NIHSS denotes National Institutes of Health Stroke Scale.

### Assessment of primary outcome

A structured telephone interview with each patient was held at 3 months by one of three trained research nurses, blinded for treatment allocation, to assess the primary outcome on the mRS. This structured telephone interview was validated in an earlier study [11].

### Assessment of secondary outcomes

The assessment of secondary outcomes will be performed as described below, for each outcome separately:

#### Infection rate during hospital admission

**Figure 2:**
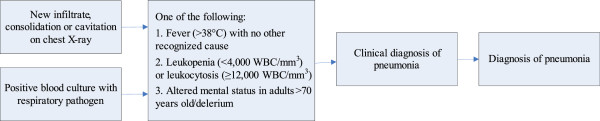
**Diagnosis of pneumonia.**

**Figure 3:**
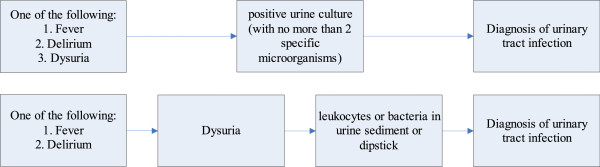
**Diagnosis of urinary tract infection.**

The total number of patients diagnosed with one or more infection(s) during hospital admission will be reported, as well as the total number of infections. Infections will be reported according to subtypes pneumonia, urinary tract infection and other infection. Infection will be assessed in two ways. First, the infection will be diagnosed in the clinical setting by the treating physician and registered as pneumonia, urinary tract infection or other infection. The clinical diagnosis of infection will be used for the primary analysis. Suspected infections without diagnostics being performed are also recorded and reported as such (for example, in a patient with a palliative care policy). Second, infection will be categorized by two infectious disease specialists who are blinded for treatment allocation, using the modified criteria of the Centers for Disease Control and Prevention (CDC criteria) [12]. For this second categorization, patients with fever, new onset delirium or clinical diagnosis of infection during hospital admission will be reviewed. For this purpose, data on the diagnostic procedures during admission as recorded in the Case Record Form (CRF) will be used. For the diagnosis of pneumonia and urinary tract infection prespecified algorithms will be used based on the CDC-criteria (Figures 2 and 3). Patients with a positive blood culture or a positive culture from the presumed site of infection, other than the lungs or urine, with a clinically relevant pathogen will be diagnosed as ‘other infection.’ Patients will be categorized as having confirmed pneumonia, urinary tract infection, or other infection. Only bacterial infections will be assessed since preventive antibiotic therapy aims to reduce these infections. Infection with *Clostridium difficile* is reported as a treatment complication. Case definition of this infection is diarrhea plus a positive *C. difficile* toxin test. Clostridium infection was diagnosed by the treating physician and was reviewed by the expert panel.

#### Death rate at discharge and at 3 months

Death during hospital admission was recorded in the CRF by the treating physician and notified as an SAE to the trial office. Death was also registered at the 3 months follow-up. If needed, survival status at 3 months was evaluated through contact with general practitioners and the municipality register.

#### Length of hospital stay

The day of admission and discharge was recorded in the CRF by the treating physician. Length of hospital admission is measured in days.

#### Total use of antibiotics during hospital stay

The use of antibiotics other than preventive antibiotic therapy will be recorded in the case record form. Total antibiotic use will be recorded in units of the ‘defined daily dose’ (DDD) and the number of days of use. For definitions of the DDD, classification according to the World Health Organization (WHO) will be used for each antibiotic [13].

#### Volume of post-stroke care, cost-effectiveness analysis

The cost-effectiveness will be measured by an economic analysis conducted alongside the study. This analysis is not included in the publication to which this analysis plan applies.

### Assessment of safety outcomes

Safety outcomes are complications of treatment, SAEs and SUSARs. All SAEs and SUSARs during the hospital stay are recorded in case record forms by the treating physician and reported to the trial office. SAEs and SUSARs occurring after discharge are recorded during the follow-up interview at 3 months. The physician records treatment complications in the CRF (diarrhea caused by *C. difficile*, allergic reaction that caused cessation of ceftriaxone, infection with ceftriaxone resistant microorganism, phlebitis at place of IV-catheter, elevation of liver enzymes, oliguria or elevation of serum creatinine).

Cause of death will be reviewed by two independent observers. They will use information from the hospital discharge letter or the medical correspondence received by the general practitioner in case the patient died after discharge. Discrepancies will be reviewed in a consensus meeting in the presence of a third investigator. Outcome parameters were derived from three recent cardiovascular trials and were modified for expected outcomes in our study [14–16]. A distinction will be made among a cardiovascular cause (brain infarction, brain hemorrhage, myocardial- or pulmonary embolism. or another cardiovascular cause), an infection (pneumonia, sepsis or another infection), death by any type of malignancy, death by any other cause (for example, traffic accident), withdrawal of treatment due to a poor prognosis or unknown cause of death.

### Analysis of primary outcome

**Figure 4:**
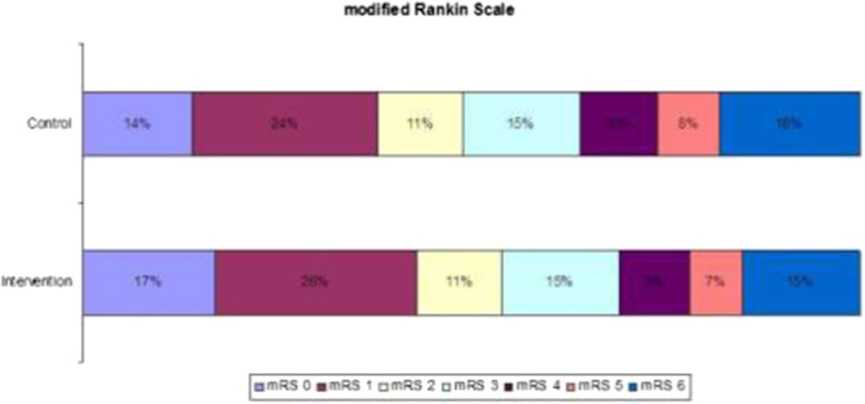
**Graphic display of primary outcome.**

An ordinal regression model on the total range of the mRS will be performed as the first analysis of primary outcome, under the assumption of proportional odds [7]. The distribution of primary outcome (for example, functional outcome on the mRS) in both treatment groups will be expressed in a histogram (Figure 4). Both adjusted and unadjusted analyses will be performed and reported. In clinical trials, adjusting for prognostic covariates improves statistical power, can correct for imbalances in baseline prognostic variables and can reduce variability in data [17, 18]. The choice of prognostic covariates is mostly based on imbalances across treatment groups, prognostic factors that are related to the primary outcome, or a combination of both [17]. As the investigators are blinded for all outcome data until the statistical analysis plan is accepted for publication, we chose to use the most important prognostic factors for outcome after stroke: age, stroke severity on the NIHSS, history of stroke, history of diabetes, prior disability as defined on mRS, and stroke type [19]. Stratification of randomization was performed according to both study center and stroke severity, so we will also include study center as a covariate. The second analysis of the primary endpoint, that is, the dichotomized score on the mRS (for example, favorable versus unfavorable, mRS 0 to 2 versus mRS 3 to 6), will be expressed as OR with 95% confidence intervals (CI; Table 4). Results of the dichotomized approach will be compared to the results of the primary analysis of primary outcome.

**Table 4:** **Secondary outcomes**

	Ceftriaxone + standard care (n = …)	Standard care (n = …)	***P***	OR 95% CI
*Secondary analysis of primary outcome*				
Favorable outcome - % n/N				
*Secondary outcomes:*				
Clinical diagnosis of infection during admission – n				
- Pneumonia				
- Urinary tract infection				
- Other				
Diagnosis of infection based on expert panel - n				
- Pneumonia				
- Urinary tract infection				
- Other				
Mortality - % n/N				
- At discharge				
- At 3 months				
Length of hospital stay - days				**NA**

NA, not applicable.

### Analysis of secondary outcomes

The number of patients with one or more in-hospital post-stroke infection(s) will be presented as numbers with event of numbers evaluated and analyzed using the chi-square test, and OR estimates with 95% CI. Infection rates will be reported as ‘judged by treating physicians’ and ‘infectious diseases panel.’ Death rate at discharge and at 3 months will also be analyzed using the chi-squared test and presented as OR estimates and 95% CI. Use of antibiotics in defined daily doses and length of hospital admission will be analyzed using the two group t-test or Mann- Whitney test where appropriate (Table 4). The analysis of volume of post-stroke care, use of antibiotics during 3 months follow-up and the cost-effectiveness analysis will be analyzed using a separate analysis protocol and presented in a subsequent paper and is, therefore, not discussed here.

### Safety outcomes

Complications of treatment, SAE’s and SUSAR’s per patient will be tabulated according to treatment group, and analyzed using the chi-squared test (Table 5 and table 6).

**Table 5:** **Number and type of serious adverse events**

Type of SAE* -% n/N	Ceftriaxone + standard care (n = …)	Standard care (n = …)	***P***
- Death			
- Life-threatening event			
- New hospitalization			
- Prolongation of existing hospitalization			
- Persistence of significant disability or incapacity			
Total number of SAE’s			

*SAE, serious adverse event.

**Table 6:** **Complications of treatment**

Adverse reaction - % n/N	Ceftriaxone + standard care (n = …)	Standard care (n = …)	***P***
- Diarrhea caused by *C. difficile*			
- Allergic reaction that caused cessation of ceftriaxone			
- Infection with ceftriaxone-resistant microorganism			
- Phlebitis at place of IV-catheter			
- Elevation of liver enzymes			
- Oliguria or elevation of serum creatinine			

Total number of adverse reactions - number %.

### Subgroup analysis of primary and secondary outcomes

We will perform the following sub-group analyses for the primary outcome: stroke type (infarction or hemorrhage), stroke severity (NIHSS 1 to 9 or NIHSS 10 to 30), time between stroke symptoms and start of the antibiotic treatment (0 to 12 h versus 12 to 24 h) and age. For the subgroup analysis of primary analysis of primary outcome, the single OR from the proportional odds model will be calculated for each subgroup separately. For the subgroup analysis of secondary analysis of primary outcome, we will tabulate the results and analyze them using the chi-squared test and presented as OR and 95% CI ((Table 7: subgroup analysis of primary outcome). In addition to these predefined subgroup analyses, we will perform a larger set of exploratory additional analyses. For secondary outcomes, we will perform all the previous mentioned subgroup analyses (stroke type, severity, time to treatment, and age). In addition we will perform analysis on presence of a swallowing disorder, respiratory tract infections, and a urinary catheter.

**Table 7:** **Subgroup analysis of primary outcome**

	Ceftriaxone + standard care (n = …)	Standard care (n = …)	***P***	OR 95% CI
Favorable outcome (mRS* 0 to 2) - % n/N				
- Ischemic stroke				
- Hemorrhagic stroke				
- Transient ischemic attack (TIA)				
- Other				
Favorable outcome (mRS 0 to 2) - % n/N				
- NIHSS** 1 to 9				
- NIHSS 10 to 30				
Favorable outcome (mRS 0 to 2) - % n/N				
- time to treatment 0 to 6 h				
- time to treatment 6 to 12 h				
- time to treatment 12 to 24 h				

*mRS, denotes modified Rankin Scale.

**NIHSS denotes National Institutes of Health Stroke Scale.

### Authorships

Two PhD students (WFW and J-DV) of this project will share first authorship; the two principle investigators (PIs) of this project will share last authorship (DvdB and PJN; DvdB corresponding author); local investigators who included at least 100 patients will be co-author; PASS study group members and physicians in expert panels for outcome-scoring will be co-author; and all local investigators who included less than 100 patients in PASS will be explicitly listed in the PASS investigators list.

### Discussion

The aim of our study is to investigate whether preventive antibiotic therapy improves functional outcome by reducing the number of infections in acute stroke patients. With this SAP, we present the analyses that will be published in the primary publication. By publishing the statistical analysis plan before knowledge of any outcome, we stimulate transparency of scientific conduct and allow others to add timely suggestions for additional analyses.

Patients in the acute phase of stroke are at risk for infections. A systematic review and meta-analysis of 87 studies showed that infections complicate stroke in 30% of all stroke patients. Pneumonia was associated with mortality with an OR of 3.62 (95% CI 2.80 to 4.68) [3]. The effect of preventive antibiotic therapy on outcome in stroke patients has been investigated in few studies. Two meta-analyses of these studies showed that preventive antibiotic therapy reduced the number of infections [4, 5]. The proportion of patients who died and the number of disabled patients were not significantly reduced, but numbers of included patients were small.

The PROBE design with open-label preventive antibiotics might introduce detection bias for infection. Physicians are aware of the treatment allocation, which potentially influences decisions on nonscheduled treatment (that is, the detection and treatment of patients with infection). This might influence the outcome measure of infection rate. To control for this bias, we will provide a secondary judgement of infection diagnosis by a blinded expert panel, according to CDC criteria. The CDC criteria are restrictive and use ancillary investigations such as blood tests, chest X-rays and culture results to confirm the diagnosis of infection. In clinical practice, for a stroke patient with fever, a cough and abnormalities on auscultation, a physician will often not wait for culture results or refrain from treating pneumonia when a chest X-ray does not (yet) show a consolidation.

Preventive treatment with ceftriaxone after stroke might improve outcome by preventing infections. A potential beneficial effect on functional outcome might be caused by a direct effect of prevention of infections in patients after stroke, most commonly pneumonia, but also by the result of decreased length of stay on the stroke unit of even in the hospital. A recent study of individual patient data in a meta-analysis of randomized trials of ventilator-associated pneumonia prevention showed that an overall attributable mortality of ventilator-associated pneumonia is 13%, which was mainly caused by prolonged exposure to the risk of dying due to increased length of ICU stay [20]. Ceftriaxone also has neuroprotective properties, at least in animal studies of stroke, which may be mediated by increased expression and activity of the glutamate transporter [21].

Antibiotics may induce overgrowth of antibiotic resistant pathogens in individual patients [22]. In the general population, selective antibiotic pressure is an important determinant of emergence and dissemination of antibiotic resistance [23, 24]. Previous clinical trials on preventive antibiotic therapy in stroke, antibiotic resistance patterns of bacteria cultured from patients with or without preventive antibiotics were similar, but numbers of patients were low [25]. Previous work has showed that implementation of preventive antibiotics in the ICU did not increase resistance rates in an environment with low levels of antibiotic resistance [26]. We will compare total antibiotic use in both treatment groups during hospital stay and collect stool specimens in a nested case control study that includes 300 patients.

During the course of the study we changed the analysis of primary outcome on the mRS from a dichotomized analysis toward an ordinal regression analysis. The ordinal regression analysis is increasingly used in stroke trials because of its higher efficiency [27]. Importantly, our primary outcome (for example, functional outcome on the mRS) was not changed, and the assumptions used in the initial sample size calculation were maintained. By using ordinal regression analysis, the total sample size was lowered from 3,200 patients to 2,550 patients. Using this method enables us to reduce the number of patients without changing the assumptions on the magnitude of the effect on the primary outcome scale from the original sample size calculation.

## Abbreviations

CRF: Case Record Form
CONSORT: Consolidated Standards of Reporting Trials
DDD: defined daily dose’
ITT: intention to treat
LOCF: last observation carried forward
mRS: modified Rankin Scale
NIHSS: National Institutes of Health Stroke Scale
OR: odds ratio
PASS: Preventive Antibiotics in Stroke Study
PP: per protocol
QALYS: quality adjusted life years
SAE: serious adverse event
SAP: statistical analysis plan
SUSAR: suspected unexpected serious adverse reaction
TIA: transient ischemic attack
VAP: ventilator-associated pneumonia.
